# Prevalence and Intensity of Catastrophic Health Expenditure Among Residents in a Multiethnic Province in China: Cross-Sectional Study

**DOI:** 10.2196/78696

**Published:** 2025-12-12

**Authors:** Wenning Sun, Shilong Zhang, Xingli Ma, Yuguo Ye, Zucheng Fang, Yuxing Zheng, Gang Cheng, Qingyue Meng, Haipeng Wang

**Affiliations:** 1 Department of Social Medicine and Health Management School of Public Health, Cheeloo College of Medicine Shandong University Jinan, Shandong China; 2 NHC Key Lab of Health Economics and Policy Research Shandong University Jinan, Shandong China; 3 Shandong Provincial Key New Think Tank Center for Health Management and Policy Research Shandong University Jinan, Shandong China; 4 Hainan Institute for Health Development Studies Haikou, Hainan China; 5 Hainan Medical University Haikou, Hainan China

**Keywords:** catastrophic health expenditure, prevalence, intensity, quantile regression, China

## Abstract

**Background:**

Hainan is a pilot free trade port in China and a multiethnic province. Catastrophic health expenditure (CHE) reflects health care inequity, particularly affecting vulnerable groups in rapidly developing multiethnic regions.

**Objective:**

This study aims to analyze CHE prevalence and intensity and their influencing factors among residents in a Chinese multiethnic province.

**Methods:**

Data from the 2023 Hainan Province Health Services Survey, conducted among 14,532 individuals aged 18 years or older, were used in a multistage stratified cluster sampling. CHE was defined as out-of-pocket health care payments exceeding 40% of the household’s capacity to pay. The chi-square test and a logistic regression were used to identify influencing factors for CHE. Nonparametric tests and quantile regression were used to evaluate the influencing factors for CHE intensity.

**Results:**

The prevalence of CHE in Hainan province was 8.37%, with a median intensity of 17.67% (IQR 8.31%-29.77%). Residents were more likely to experience CHE if they were older than 60 years (odds ratio [OR] 1.928, 95% CI 1.602-2.320; *P*<.001), unmarried (OR 1.241, 95% CI 1.075-1.433; *P*=.003), or had chronic illnesses (OR 2.214, 95% CI 1.930-2.540; *P*<.001). Ethnic minority groups (OR 0.774, 95% CI 0.679-0.883; *P*<.001) as well as middle-income (OR 0.722, 95% CI 0.600-0.869; *P*=.001), high-middle-income (OR 0.739; 95% CI 0.609-0.898; *P*=.002), and high-income (OR 0.591, 95% CI 0.474-0.738; *P*<.001) groups were less likely to experience CHE. At lower CHE intensity (20th percentile), individuals older than 60 years (β=1.935; *P*=.03) and middle-income (β=1.737; *P*=.04) and rural (β=2.202; *P*=.005) residents showed positive associations. At the 50th percentile, low-middle-income (β=–5.052; *P*=.005), high-middle-income (β=–4.203; *P*=.03), and high-income (β=–6.534; *P*=.004) groups showed negative associations. At the 80th percentile, high-middle-income (β=–7.143; *P*=.03) and rural (β=–6.241; *P*=.005) groups showed stronger financial protection.

**Conclusions:**

Addressing CHE risks remains a critical challenge in Hainan province, highlighting structural inequities rooted in socioeconomic disparities and health vulnerabilities. Therefore, policy should prioritize primary prevention in the lowest-income populations while implementing enhanced insurance coverage for rural populations facing extreme costs to alleviate the most severe financial burdens.

## Introduction

Universal health coverage (UHC) aims to provide everyone with quality health services while ensuring they do not incur financial hardship [1]. However, high out-of-pocket (OOP) health expenditures can hinder the achievement of UHC and create a vicious cycle of poverty and poor health [2]. Catastrophic health expenditure (CHE) is defined as annual OOP health care payments exceeding a predetermined percentage of the household capacity to pay, which serves as a critical indicator for monitoring UHC progress and health care inequity [3]. Globally, approximately 1.4 billion people experience CHE annually, with more than 60% residing in low- and middle-income countries [4]. As the world’s largest middle-income country, China faces significant challenges in reducing CHE, with an incidence of 8.70% in 2018 [5]. Therefore, addressing CHE deserves heightened attention and strategic efforts, which are pivotal for reducing health inequalities and mitigating poverty.

Globally, efforts to mitigate CHE have focused on strengthening financial protection through UHC [6]. The World Health Organization advocates reducing financial barriers to health care access for vulnerable populations, adopting legislation to prevent medical impoverishment, and establishing health financing mechanisms [7]. In China, the health care reform that began in 2009 expanded basic medical insurance, which now covers more than 95% of the population [8]. The Healthy China 2030 plan further institutionalizes payment reforms and centralized procurement of drugs [9,10]. However, considering only the national achievements may mask regional disparities [11]. Existing studies have demonstrated significant variations in the prevalence of CHE across Chinese provinces, with the highest value differing by up to 2.5 times from the lowest [11,12].

CHE is influenced by multiple factors, such as economic condition, residence, ethnicity, chronic diseases, and age [13]. Among them, economic condition and ethnicity are important explanatory variables affecting CHE [14,15]. Most studies indicate that ethnic minority groups are one of the most vulnerable groups in terms of health and medical cost burden [16,17]. Among Native Americans, low health insurance coverage and high-cost diseases, such as diabetes, often make health expenditure a major financial burden [18]. In addition, studies highlight that rapid economic development has exacerbated CHE, particularly in emerging markets, such as Brazil, China, and India [14,19]. The rapid economic development brings many challenges, including the large income disparities, lagging social security system, and uneven distribution of medical resources, all of which have been proven to increase economic inequality in health care [20,21].

Existing research on CHE has focused on national-level insights, examining the economic burdens of older individuals, specific diseases, and rural regions [13,22,23]. Cross-national studies have also identified systemic drivers, notably insurance fragmentation [24,25]. Although some studies have examined provincial-level differences, few have investigated regions with specific economic and ethnic structures, especially those undergoing rapid economic development [11,15].

Hainan province, located in the southernmost part of China, is the only tropical island province in the country, with a relatively small and dispersed population. Ethnic minority groups account for 15.7% of the population, significantly higher than the national average (8.9%), with the Li ethnic group being the main ethnic minority group. Economically, this province ranks fourth from the bottom in terms of gross domestic product within the country. Region-specific results are crucial for shaping effective health care policies, as they enable the alignment of public health investments with localized needs. This study aims to analyze the prevalence and intensity of CHE as well as their influencing factors among residents in Hainan province, a free trade port with a multiethnic population. The findings of this research are expected to inform the design of targeted strategies, ultimately contributing to the reduction of health inequalities in multiethnic areas with rapid economic development.

## Methods

### Data Source

Data for this cross-sectional study were obtained from the 2023 Hainan Province Health Services Survey. The survey was organized by the Hainan Provincial Health Commission and was a part of the China National Health Services Survey, which has been conducted every 5 years since 1993. The data are representative of Hainan province in terms of geographic location, level of economic development, and population distribution characteristics. A multistage stratified cluster sampling method was used to conduct the survey. All cities, counties, and autonomous counties in Hainan province were included in the scope of this survey. A total of 24 districts and counties were sampled. Within each district or county, 2 to 5 towns (or streets) were randomly selected, and within each selected town (or street), 2 villages (or communities) were further randomly chosen. Subsequently, within each selected village (or community), 60 households were randomly selected. A standardized questionnaire was used to collect data from all residents in the sampled households. In total, 6485 households, with a population of 18,677, were surveyed.

Trained interviewers conducted household visits and face-to-face interviews with all household members using mobile phones or tablets equipped with an electronic survey system. Each resident was individually questioned according to the survey’s electronic questionnaire. The questionnaire collected information on demographic and socioeconomic characteristics, household expenditures, health status, insurance coverage, and health service use. The study population consisted of residents of Hainan province aged 18 years or older. After excluding missing value samples, 14,532 adult participants were included.

### Ethical Considerations

The 2023 Hainan Province Health Services Survey (organized by the Hainan Provincial Health Commission) was approved by the institutional review board of the National Bureau of Statistics of China. This study is part of the National Health Service Survey (NHSS), a nationwide survey conducted across all 31 provinces of China under the authority of the National Bureau of Statistics (NBS). The 2023 Hainan Health Service Survey represents the province-level extension undertaken by the Hainan Health Commission within the framework of the NHSS. Ethical review for the NHSS is provided through the institutional review procedures of the NBS. As is standard for national statistical investigations in China, the internal approval reference numbers are not publicly disseminated, but the survey is widely recognized as a routine, government-mandated activity with established ethical oversight. Oral informed consent was obtained from all participants before the interview. The survey was conducted anonymously, and all data were deidentified and kept confidential. Participation was voluntary, and no compensation was provided to participants. All procedures were conducted in accordance with the Declaration of Helsinki and relevant ethical guidelines.

### CHE Measurement

The threshold of CHE is calculated as the proportion of medical expenses to household income or expenditure (excluding food expenditure) [26]. The selection of thresholds for defining CHE varies from 2.5% to 60%, with the commonly used criterion in studies conducted in low- and middle-income countries being the 40% threshold recommended by the World Health Organization [27]. We adopted a threshold of CHE at 40% of a household’s total nonfood expenditure for self-paid medical expenses in the year 2022. If the expenditure was below this threshold, it was considered that CHE had not occurred. The intensity of CHE was defined as the average extent to which households exceeded the catastrophic threshold, calculated among all households whose medical expenditure share surpassed the 40% cutoff. [28]. The total nonfood expenditure of a household is the difference between the total household expenditure and the household’s food expenditure. In addition, we included individual participants in our analysis, rather than the households they belonged to, as this approach allowed us to consider the individual characteristics of the residents. OOP medical expenses were measured by asking “What was your household’s expenditure on medical care in 2022?” The total household expenditure was measured by asking “What was your household’s total consumption expenditure in 2022, approximately in yuan?” The household’s food expenditure was measured by asking “How much did your household spend on food, tobacco, and alcohol in 2022?”

### General Demographic Information

General demographic information included sex (male or female), ethnic group (Han ethnicity or ethnic minority groups), age (18-39 y, 40-59 y, or 60 y), marital status (married or unmarried), educational level (illiterate or semiliterate, primary school, junior school, senior high school, or above), residence (urban or rural), medical insurance (Urban Employee Basic Medical Insurance, Urban and Rural Residents’ Basic Medical Insurance, or others), annual income quintiles (low, low middle, middle, high middle, or high), and chronic disease (no or yes). National civil servants, professionals, enterprise managers, and active military personnel were classified as senior practitioners based on their professional levels and required skills; clerks and workers were classified as midlevel practitioners; and farmers, freelancers, self-employed individuals, unemployed persons, and students were classified as general staff. The annual income quintile referred to the division of an individual’s personal annual income or the per capita annual income of their household into 5 equal parts.

### Statistical Analysis

We used frequencies (n) and percentages (%) to depict the sociodemographic and CHE-related characteristics of residents in Hainan province. The prevalence of CHE was calculated as the proportion of respondents who experienced CHE among all survey participants. On the basis of the chi-square test, we compared the categorical factors associated with CHE. Dummy variables were set for the categorical variables with more than 2 groups. In the multivariate analysis, binary logistic regression was performed to examine the factors associated with CHE, as the dependent variable, indicating whether the respondent experienced CHE, was dichotomous (yes or no). The odds ratios (ORs), 95% CIs, and *P* values were reported. The intensity of CHE showed a significantly skewed distribution; therefore, medians and IQRs were reported. The Mann-Whitney rank-sum test was used for 2-group comparisons, and the Kruskal-Wallis test was used for multiple comparisons. Quantile regression was used to explore the influencing factors of the intensity of CHE at different levels (20th, 50th, and 80th percentiles) among residents who had incurred CHE. All statistical analyses were conducted using Stata (version 16.0; StataCorp). The significance level for statistics was set at a 2-sided *P*<.05.

## Results

### Basic Characteristics of the Participants

The characteristics of the participants are shown in Table 1. A total of 14,532 participants were enrolled in this study, of whom 41.74% (n=6065) belonged to ethnic minority groups. The overwhelming majority of participants were male (n=7863, 54.11%), aged 40 to 59 years (n=6043, 41.58%), married (n=10,941, 75.29%), with junior school education (n=6030, 41.49%), were general staff (n=11,502, 79.15%), from rural areas (n=8876, 61.08%), had Urban and Rural Residents’ Basic Medical Insurance (n=11,473, 78.95%), and did not have chronic disease (n=11,564, 79.58%). In addition, the annual income quintiles were divided into 5 groups from low to high.

**Table 1:** Prevalence of catastrophic health expenditure (CHE) by baseline characteristics.

Characteristic		Participants, n/N (%)			CHE=no, n/N (%)	CHE=yes, n/N (%)			Chi-square (*df*)			*P* value	
Total		14,532/14,532 (100)			13,316/14,532 (91.63)	1216/14,532 (8.37)			—^a^			—	
**Sex**										0.1 (1)			.81
	Male		7863/14,532 (54.11)	7209/7863 (91.68)			654/7863 (8.32)						
	Female		6669/14,532 (45.89)	6107/6669 (91.57)			562/6669 (8.43)						
**Ethnic group**										10.2 (1)			.001
	Han ethnicity		8467/14,532 (58.26)	7706/8467 (91.01)			761/7706 (8.99)						
	Ethnic minority groups		6065/14,532 (41.74）	5610/6065 (92.5)			455/6065 (7.5)						
**Age (y)**										275.3 (2)			<.001
	18-39		4598/14,532 (31.64)	4338/4598 (94.35)			260/4598 (5.65)						
	40-59		6043/14,532 (41.58)	5657/6043 (93.61)			386/6043 (6.39)						
	≥60		3891/14,532 (26.78)	3321/3891 (85.35)			570/3891 (14.65)						
**Marital status**										4.5 (1)			.034
	Married		10,941/14,532 (75.29)	10,056/10,941 (91.91)			885/10,941 (8.09)						
	Unmarried		3591/14,532 (24.71)	3260/3591 (90.78)			331/3591 (9.22)						
**Educational level**										58.9 (3)			<.001
	Illiterate or semiliterate		1060/14,532 (7.29)	921/1060 (86.89)			139/1060 (13.11)						
	Primary school		3522/14,532 (24.24)	3179/3522 (90.26)			343/3522 (9.74)						
	Junior school		6030/14,532 (41.49)	5551/6030 (92.06)			479/6030 (7.94)						
	Senior high school or above		3920/14,532 (26.97)	3665/3920 (93.49)			255/3920 (6.51)						
**Careers**										16.9 (2)			<.001
	General staff		11,502/14,532 (79.15)	10,484/11,502 (91.15)			1018/11,502 (8.85)						
	Midlevel practitioners		2425/14,532 (16.69)	2269/2425 (93.57)			156/2425 (6.43)						
	Senior practitioners		605/14,532 (4.16)	563/605 (93.06)			42/605 (6.94)						
**Annual income quintiles**										38.3 (4)			<.001
	Low		2864/14,532 (19.71)	2557/2864 (89.28)			307/2864 (10.72)						
	Low middle		3434/14,532 (23.63)	3126/3434 (91.03)			308/3434 (8.97)						
	Middle		2943/14,532 (20.25)	2715/2943 (92.25)			228/2943 (7.75)						
	High middle		2679/14,532 (18.44)	2474/2679 (92.35)			205/2679 (7.65)						
	High		2612/14,532 (17.97)	2444/2612 (93.57)			168/2612 (6.43)						
**Residence**										7.7 (1)			.005
	Urban		5656/14,532 (38.92)	5228/5656 (92.43)			428/5656 (7.57)						
	Rural		8876/14,532 (61.08)	8088/8876 (91.12)			788/8876 (8.88)						
**Medical insurance**										13.7 (2)			.001
	Urban Employee Basic Medical Insurance		2321/14,532 (15.97)	2168/2321 (93.41)			153/2321 (6.59)						
	Urban and Rural Residents’ Basic Medical Insurance		11,473/14,532 (78.95)	10,463/11,473 (91.2)			1010/11,473 (8.8)						
	Others		738/14,532 (5.08)	685/738 (92.82)			53/738 (7.18)						
**Chronic disease**										298.9 (1)			<.001
	No		11,564/14,532 (79.58)	10829/11,564 (93.64)			735/11,564 (6.36)						
	Yes		2968/14,532 (20.42)	2487/2968 (83.79)			481/2968 (16.21)						

^a^Not applicable.

### Prevalence of CHE by Basic Characteristics

Table 1 shows the prevalence of CHE and disparities in basic characteristics among the residents of Hainan province. Among 14,532 participants, the prevalence of CHE was 8.37%. As the results of the chi-square test revealed, CHE was significantly associated with ethnic group (*χ*^2^_1_=10.2; *P*=.001), age (*χ*^2^_2_=275.3; *P*<.001), marital status (*χ*^2^_1_=4.5; *P*=.03), educational levels (*χ*^2^_3_=58.9; *P*<.001), careers (*χ*^2^_2_=16.9; *P*<.001), annual income (*χ*^2^*_4_*=38.3; *P*<.001), residence (*χ*^2^_1_=7.7; *P*=.005), medical insurance (*χ*^2^_2_=13.7; *P*=.001) and chronic disease status (*χ*^2^_1_=298.9; *P*<.001).

### Determinants of CHE Identified by Logistic Regression

Table 2 shows the results of logistic regression analysis of the determinants of CHE. Ethnic minority residents were less likely to have experienced CHE than residents from the Han community (OR 0.774, 95% CI 0.679-0.883; *P*<.001). Compared to residents aged between 18 and 39 years, those aged 60 years and older were more prone to having experienced CHE (OR 1.928, 95% CI 1.602-2.320; *P*<.001). Unmarried residents were more likely to have faced CHE than married residents (OR 1.241, 95% CI 1.075-1.433; *P*=.003). In comparison to low-income groups, middle-income (OR 0.722, 95% CI 0.600-0.869; *P*=.001), high-middle-income (OR 0.739, 95% CI 0.609-0.898; *P*=.002), and high-income groups (OR 0.591, 95% CI 0.474-0.738; *P*<.001) were less likely to have encountered CHE. Residents with chronic diseases were more likely to have experienced CHE than those without chronic diseases (OR 2.214, 95% CI 1.930-2.540; *P*<.001).

**Table 2:** Determinants of catastrophic health expenditure based on logistic regression (N=14,532).

Characteristic		Odds ratio (95% CI)	*P* value
**Sex (reference=male)**			
	Female	0.949 (0.837-1.076)	.42
**Ethnic group (reference=Han ethnicity)**			
	Ethnic minority groups	0.774 (0.679-0.883)	<.001
**Age (y; reference=18-39 y)**			
	40-59	1.017 (0.851-1.217)	.85
	≥60	1.928 (1.602-2.320)	<.001
**Marital status (reference=married)**			
	Unmarried	1.241 (1.075-1.433)	.003
**Educational level (reference=illiterate)**			
	Primary school	0.989 (0.791-1.235)	.92
	Junior school	0.977 (0.778-1.227)	.84
	Senior high school or above	0.842 (0.650-1.090)	.19
**Careers (reference=general staff)**			
	Midlevel practitioners	0.900 (0.709-1.144)	.39
	Senior practitioners	1.020 (0.689-1.511)	.92
**Annual income quintiles (reference=low)**			
	Low middle	0.855 (0.721-1.014)	.07
	Middle	0.722 (0.600-0.869)	.001
	High middle	0.739 (0.609-0.898)	.002
	High	0.591 (0.474-0.738)	<.001
**Residence (reference=urban)**			
	Rural	1.043 (0.912-1.193)	.54
**Medical insurance (reference=** **Urban Employee Basic Medical Insurance** **)**			
	Urban and Rural Residents’ Basic Medical Insurance	1.086 (0.831-1.419)	.55
	Others	0.820 (0.562-1.197)	.30
**Chronic disease (reference=no)**			
	Yes	2.214 (1.930-2.540)	<.001

### Intensity of CHE by Baseline Characteristics

Table 3 shows the intensity of CHE and disparities in the basic characteristics of Hainan residents. Among 1216 participants with CHE, the median intensity of CHE was 17.67% (IQR 8.31%-29.77%). The Mann-Whitney rank-sum test and Kruskal-Wallis test results show that the intensity of CHE was significantly associated with age (H=7.813; *P*=.02), careers (H=6.350; *P*=.04), and annual income quintiles (H=11.460; *P*=.02).

**Table 3:** Intensity of catastrophic health expenditure (CHE) by baseline characteristics (n=1216).

Characteristics		CHE intensity (%), median (IQR)	Statistics		*P* value	
**Sex**				Z=0.030		.98
	Male	17.47 (8.31-29.77)				
	Female	17.83 (8.31-29.77)				
**Ethnic group**				Z=1.184		.24
	Han ethnicity	19.00 (8.54-30.09)				
	Ethnic minority groups	17.14 (7.94-28.62)				
**Age (y)**				H=7.813		.02
	18-39	17.14 (6.88-27.29)				
	40-59	15.56 (7.39-29.90)				
	≥60	20.08 (9.38-30.67)				
**Marital status**				Z=0.044		.97
	Married	17.31 (8.31-29.93)				
	Unmarried	18.06 (8.19-28.77)				
**Educational level**				H=0.763		.86
	Illiterate or semiliterate	18.82 (8.31-29.93)				
	Primary school	16.60 (7.62-29.77)				
	Junior school	18.37 (8.39-29.77)				
	Senior high school or above	19.00 (8.18-29.48)				
**Careers**				H=6.350		.04
	General staff	18.39 (8.39-29.77)				
	Midlevel practitioners	16.71 (8.14-30.60)				
	Senior practitioners	10.00 (5.28-24.00)				
**Annual income quintiles**				H=11.460		.02
	Low	21.54 (8.78-30.42)				
	Low middle	16.03 (7.97-30.47)				
	Middle	19.74 (8.78-33.43)				
	High middle	17.14 (9.38-26.23)				
	High	13.58 (5.28-29.77)				
**Residence**				Z=–0.674		.50
	Urban	17.47 (6.50-34.60)				
	Rural	18.06 (8.78-29.52)				
**Medical insurance**				H=1.079		.58
	Urban Employee Basic Medical Insurance	16.24 (7.44-29.77)				
	Urban and Rural Residents’ Basic Medical Insurance	18.04 (8.32-29.77)				
	Others	20.24 (8.26-27.13)				
**Chronic disease**				Z=–1.820		.07
	No	16.60 (8.32-27.72)				
	Yes	20.39 (8.18-31.57)				

^a^For the total population, the median CHE intensity was 17.67 (IQR 8.31-29.77).

### Determinants of the Intensity of CHE Identified by Quantile Regression

Table 4 shows the results of quantile regression for the determinants of the intensity of CHE. The regression results at lower quantile points indicated that residents aged 60 years and older were associated with an increase in the intensity of CHE (β=1.935; *P*=.04). In the presented quantile regression results, annual income quintiles were factors influencing the intensity of CHE, except for varying directions of their impacts. At lower quantile points, the regression results showed that middle income was associated with an increase in the intensity of CHE (β=1.737; *P*=.05). The regression results at medium quantile points indicated that low-middle income (β=–5.052; *P*=.01), high-middle income (β=–4.203; *P*=.05), and high income (β=–6.534; *P*=.01) were all associated with a decrease in the intensity of CHE. The regression results at higher quantile points indicated that high-middle income was associated with a decrease in the intensity of CHE (β=–7.143; *P*=.03). Both the regression results at lower and higher quantile points indicated urban-rural differences as a factor influencing the intensity of CHE. Notably, in the regression at lower quantile points, residence in rural areas was associated with an increase in the intensity of CHE (β=2.202; *P*=.001), whereas at higher quantile points, it was associated with a decrease in the intensity of CHE (β=–6.241; *P*=.007).

**Table 4:** Determinants of the intensity of catastrophic health expenditure based on quantile regression (n=1216).

Characteristic			20th percentile					50th percentile					80th percentile		
			β		*P* value		β			*P* value		β			*P* value
**Sex (reference=male)**															
	Female	0.012		.98		0.460			.74		–1.472			.49	
**Ethnic group (reference=Han ethnicity)**															
	Ethnic minority groups	–0.162		.80		–1.630			.25		–2.846			.20	
**Age (y; reference=18-39 y)**															
	40-59	0.491		.59		0.368			.86		0.153			.96	
	≥60	1.935		.04		3.254			.12		–0.147			.96	
**Marital status (reference=married)**															
	Unmarried	0.084		.90		1.668			.30		–1.941			.43	
**Educational level (reference=illiterate)**															
	Primary school	–0.146		.89		–0.146			.95		–4.075			.26	
	Junior school	0.830		.43		1.929			.42		–3.741			.31	
	Senior high school or above	1.467		.22		2.023			.46		–4.744			.26	
**Careers (reference=general staff)**															
	Midlevel practitioners	–0.212		.86		–0.370			.89		3.215			.44	
	Senior practitioners	–1.610		.41		–7.430			.10		–4.637			.50	
**Annual income quintiles (reference=low)**															
	Low middle	0.888		.27		–5.052			.01		–2.796			.33	
	Middle	1.737		.05		–2.642			.19		1.499			.63	
	High middle	1.699		.07		–4.203			.05		–7.143			.03	
	High	–1.236		.26		–6.534			.01		–2.542			.51	
**Residence (reference=urban)**															
	Rural	2.202		.001		–0.357			.81		–6.241			.007	
**Medical insurance (reference=Urban Employee Basic Medical Insurance)**															
	Urban and Rural Residents’ Basic Medical Insurance	–1.347		.31		–0.567			.85		–0.398			.93	
	Others	–2.168		.24		–1.257			.76		–2.673			.68	
**Chronic disease (reference=no)**															
	Yes	–0.711		.27		2.403			.10		2.424			.28	

## Discussion

### Principal Findings

This study found that the prevalence of CHE in Hainan province was 8.37%, indicating that nearly one-tenth of the residents experienced financial hardship when seeking health care. When using the same calculation method and threshold, this provincial prevalence aligns with China’s national estimates of 8.70% in 2018 but exceeds reported figures from Malaysia (2.80% in 2016) and Pakistan (4.57% in 2019) [5,29,30]. The intensity of CHE in Hainan province (17.67%) further highlights the severe economic burden, surpassing the intensity in Iran (12.88% in 2015) and Bhutan (0.80% in 2017) [31,32]. The high prevalence and intensity of CHE in Hainan province may correlate with China’s low overall welfare level, as nationwide OOP health care payments reach 28%, far exceeding the international benchmark of 20% [5,33]. Notably, the development of the Hainan Free Trade Port has greatly increased the risk of CHE. Tourism expansion and seasonal migrants may exacerbate the strain on health care resources, inflating service prices and increasing economic burdens [34]. Concurrently, the migrant population (eg, flexible workers) demonstrates heightened financial vulnerability due to interregional barriers to medical expense reimbursement [35]. Therefore, there is significant room for improvement in the control of health expenditures in Hainan province.

Contrary to previous evidence that minority groups are at higher risk of CHE (eg, the US Hispanic population), ethnic minority groups in Hainan province exhibit lower CHE prevalence than Han residents [18]. This phenomenon reflects a distinct sociocultural context in Hainan province. Ethnic minority groups benefit from preferential health care subsidies that are channeled to autonomous regions, directly lowering OOP medical costs [36]. Complementing this, traditional ethnic villages maintain mutual-aid financing systems that buffer against sudden health care expenses by pooling resources to share financial risks [37]. Meanwhile, ancestral medical practices, such as fire needle therapy and herbal poultices preserved by the Li and Miao ethnic groups, decrease reliance on costly modern treatments, further mitigating the likelihood of CHE [38]. In contrast, urbanized Han populations rely heavily on expensive fee-for-service tertiary hospitals, thereby exacerbating financial burdens [17]. In addition, given that ethnic minority groups generally have lower family costs compared to the Han population in China, it seems less likely that family cost is a major contributing factor to the lower prevalence of CHE in this population [39,40]. Therefore, special subsidy criteria for diseases prevalent among ethnic minority groups (such as areca oral cancer screening) should be refined. It is also important to accelerate the inclusion of Li and Miao traditional medicines in the insurance catalog.

This study revealed the subtle relationship between residence and CHE intensity in Hainan province. At the lower quantile, rural areas were associated with an increase in CHE intensity. Under the free trade policies, advanced medical resources are concentrated in urban centers, such as Haikou and Sanya [20]. Rural patients’ cross-city medical care overlaps with hidden costs, such as transportation and missed work, triggering family financial collapse [41]. Paradoxically, at the higher quantile, rural residents showed a protective effect. Possible explanations suggest that rural residents, constrained by lower economic resilience, tend to delay medical consultations, reduce treatment frequency, and prefer low-cost alternatives when facing the high costs of critical illnesses [42]. This behavioral pattern potentially exacerbates health inequalities through untreated disease progression and accumulated complications. Therefore, priority should be given to strengthening rural primary health care capacity through 5G telemedicine, incentives for family physicians, and hierarchical medical systems while establishing targeted medical relief funds for rural families [43].

Consistent with previous studies [44], income was negatively associated with experiencing CHE. However, our study revealed a complex intensity gradient in Hainan province. Specifically, middle-income residents demonstrated higher CHE intensity at lower expenditure quantiles. The sandwich-class population, while exceeding the poverty threshold, demonstrated constrained financial resilience due to inadequate social safety nets and elevated living expenditures, such as mortgage obligations [45]. In contrast, the poorest population exhibited suppressed health care demand due to chronic poverty, weakening the CHE effect [42]. Upper-middle-income groups at higher quantiles effectively mitigate health risks through commercial insurance and precautionary savings [46]. These stratified patterns highlight limitations of uniform provincial insurance policies in addressing heterogeneous income gradients. It is recommended to strengthen critical illness risk pooling mechanisms for middle-income populations, broaden medical assistance for the most impoverished, and explore internationally aligned commercial health insurance services via the free trade port of Hainan province.

Research found that age, chronic diseases, and marital status significantly impacted the likelihood of experiencing CHE, consistent with previous studies [47,48]. Older adults with chronic diseases showed higher CHE prevalence. Aging often leads to declining physical functions, while chronic diseases require long-term treatment and multiple medications, increasing cumulative health care costs [49]. Unmarried individuals had higher CHE rates than their married counterparts. This discrepancy may stem from family risk-sharing mechanisms, where married people use household financing and mutual care to buffer medical shocks, whereas unmarried individuals rely more on personal savings [50]. To address these challenges, Hainan province pioneered the “2+3” health care package, implementing 5 high-burden chronic disease screening and management for early cost control. Meanwhile, vulnerable populations, such as older individuals living alone, receive dual teams of family physicians and social workers, preventing cost escalation from delayed care. This integrated strategy offers a replicable model for developing regions.

### Limitations

This study has several limitations. First, despite our efforts to assist patients in their recall to minimize estimation error, self-reported expenditures on health care and food are subject to recall bias. Second, the data do not capture individuals who either did not seek treatment or abandoned it due to financial constraints. This may cause the research findings to underestimate the prevalence and intensity of CHE to a certain extent. Finally, this study was exclusively conducted among residents in Hainan province, China. The applicability of the findings to other regions remains unsubstantiated and will necessitate further investigation in future research.

### Conclusions

Addressing CHE risks remains a critical challenge for health systems in Hainan province. Residents aged 60 years and older, belonging to the Han ethnicity, in the lowest income quintile, who were unmarried, and with chronic conditions, exhibited a significantly higher likelihood of being confronted with CHE. Annual income, age, and residence were identified as factors influencing the intensity of CHE. It is worth noting that the direction of their influence was found to vary across the levels of the intensity of CHE. Policy should prioritize primary prevention to reduce CHE prevalence, such as chronic disease management and expanding financial protection for the older population and the poorest. Simultaneously, secondary measures, potentially through enhanced insurance coverage for specific rural populations or conditions leading to extreme costs, are needed to alleviate the highest levels of financial burden.

## Abbreviations

CHE: catastrophic health expenditure
OOP: out-of-pocket
OR: odds ratio
UHC: universal health coverage

## Data Availability

The data are managed by the Statistical Information Center of the Hainan Provincial Health Commission and are not publicly available due to institutional restrictions. However, the data used and analyzed during the study are available from the corresponding author on reasonable request.
